# 1941. Coadministration of Bivalent Respiratory Syncytial Virus (RSVpreF) Vaccine With Influenza Vaccine in Older Adults

**DOI:** 10.1093/ofid/ofad500.095

**Published:** 2023-11-27

**Authors:** James A Baber, Karen Quan, Eugene Athan, Robert Scott, Anna Jaques, Qin Jiang, Wen Li, David Cooper, Mark W Cutler, Elena Kalinina, Annaliesa S Anderson, Kena A Swanson, William C Gruber, Alejandra C Gurtman, Beate Schmoele-Thoma

**Affiliations:** Pfizer Vaccine Clinical Research, Sydney, New South Wales, Australia; Pfizer Vaccine Clinical Research, Sydney, New South Wales, Australia; Barwon Health, Geelong, Victoria, Australia; University of the Sunshine Coast, Morayfiled, Queensland, Australia; Pfizer Vaccine Clinical Research, Sydney, New South Wales, Australia; Pfizer, Collegeville, Pennsylvania; Pfizer Vaccine Clinical Research, Sydney, New South Wales, Australia; Pfizer, Collegeville, Pennsylvania; Pfizer Inc, Pearl River, New York; Pfizer, Collegeville, Pennsylvania; Pfizer, Collegeville, Pennsylvania; Pfizer, Collegeville, Pennsylvania; Pfizer, Collegeville, Pennsylvania; Pfizer, Collegeville, Pennsylvania; Pfizer, Collegeville, Pennsylvania

## Abstract

**Background:**

Respiratory syncytial virus (RSV) is an important cause of severe lower respiratory tract illness in older adults. RSV and influenza are both typically seasonal diseases, with peaks during winter in temperate climates. Annual vaccination with high-dose or adjuvanted influenza vaccine formulations is recommended to prevent influenza illness in older adults. Therefore, in a Phase 3 study in older adults, the safety and immunogenicity of coadministration of Pfizer’s investigational bivalent, stabilized RSV prefusion F subunit vaccine (RSVpreF) and adjuvanted quadrivalent seasonal inactivated influenza vaccine (SIIV) was compared with SIIV alone or RSVpreF alone.

**Methods:**

The primary immunogenicity objective of this Phase 3, 1:1 randomized, parallel-group, double-blind, placebo-controlled study with 1471 healthy adults ≥65 years of age in Australia was to demonstrate noninferiority of coadministration of RSVpreF 120 µg with SIIV compared with sequential administration of SIIV followed by RSVpreF one month later, using a 1.5-fold equivalence margin.

The safety profile of coadministration of RSVpreF with SIIV was evaluated by collection of reactogenicity and adverse events.

Study Design Schema

**Figure:**
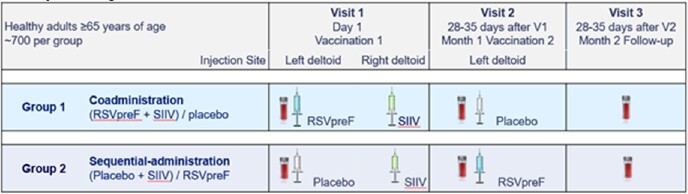

**Results:**

A total of 1403 participants were randomized, with 1399 participants who received vaccination at a median age of 70 years (range 65-91 years) included in the safety population. Local reactions and systemic events were mostly mild or moderate when RSVpreF was coadministered with SIIV. There were no vaccine related serious adverse events reported.

The primary immunogenicity objective was met. The geometric mean ratios (GMRs) ranged from 0.85 to 0.86 for RSVpreF and 0.77 to 0.90 for SIIV, for RSV neutralizing titers and strain-specific hemagglutination inhibition assay (HAI) titers at 1 month after vaccination, achieving the 1.5-fold prespecified non-inferiority margin (lower bound CI > 0.667).

Geometric Mean Titer (GMT) Ratios for RSV Neutralizing Titers (NT) and Influenza HAI titers with 95% Confidence Intervals (CI)

**Figure:**
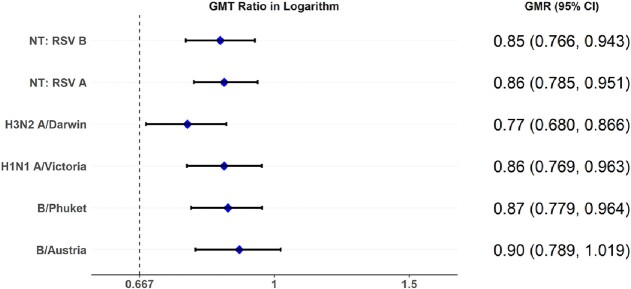

**Conclusion:**

The primary objectives of the study were met demonstrating noninferiority of the RSVpreF and SIIV immune responses when RSVpreF was coadministered with SIIV, and that RSVpreF had an acceptable safety profile when coadministered with SIIV. The results of this study support the coadministration of RSVpreF and SIIV in an older adult population.

**Disclosures:**

**James A. Baber, MBChB, MPH**, Pfizer: Salary|Pfizer: Stocks/Bonds **Karen Quan, BSc, MBBS, DPM**, Pfizer: Employee|Pfizer: Stocks/Bonds **Anna Jaques, MPH**, Pfizer: Salary|Pfizer: Stocks/Bonds **Qin Jiang, PhD**, Pfizer: Employee|Pfizer: Employee|Pfizer: Stocks/Bonds|Pfizer: Stocks/Bonds **Wen Li, PhD**, Pfizer: Stocks/Bonds **David Cooper, PhD**, Pfizer, Inc.: Stocks/Bonds **Mark W. Cutler, PhD**, Pfizer: Employee|Pfizer: Stocks/Bonds **Elena Kalinina, PhD**, Pfizer: Pfizer employee|Pfizer: Stocks/Bonds **Annaliesa S. Anderson, PhD**, Pfizer: Employee|Pfizer: Stocks/Bonds **Kena A. Swanson, Ph.D.**, Pfizer: Employee|Pfizer: Stocks/Bonds **William C. Gruber, MD**, Pfizer, Inc.: Employee|Pfizer, Inc.: Stocks/Bonds **Alejandra C. Gurtman, M.D**, Pfizer: Employee|Pfizer: Stocks/Bonds **Beate Schmoele-Thoma, MD**, Pfizer: Stocks/Bonds

